# Artificial Intelligence for Spatial Immunometabolic Analysis of the Tumor Microenvironment: Current Evidence and Future Directions

**DOI:** 10.3390/cimb48050476

**Published:** 2026-05-03

**Authors:** Ismail Abdullah, Shady Saud Khan, Sariya Khan, Dana Abou, Jana Khan, Fayza Akil, Noha Farag, Abdullah Almilaibary

**Affiliations:** 1 College of Medicine, Alfaisal University, Riyadh 11533, Saudi Arabia; iaabdullah@alfaisal.edu; 2 General Medicine Practice Program, Batterjee Medical College, Jeddah 21442, Saudi Arabia; shadysaudk@gmail.com (S.S.K.); janasaud6@hotmail.com (J.K.); fayzaakil4@gmail.com (F.A.); 3 Pharmacy Program, Batterjee Medical College, Jeddah 21442, Saudi Arabia; danaabou13@gmail.com; 4 Clinical Pathology, General Medicine Program, Batterjee Medical College, Jeddah 21442, Saudi Arabia; noha.farag@bmc.edu.sa; 5 Family and Community Medicine Department, Faculty of Medicine, Al-Baha University, Al Baha 65511, Saudi Arabia

**Keywords:** tumor microenvironment, artificial intelligence, spatial transcriptomics

## Abstract

The tumor microenvironment [TME] is a dynamic ecosystem where spatial organization and metabolic reprogramming play a crucial role in immune response, tumor progression, and therapeutic response. Recent breakthroughs in spatial transcriptomics, metabolomics, and multiplexed imaging studies have shown that complex immunometabolic niches are involved in therapeutic resistance, including conventional and immunotherapeutic approaches. Artificial intelligence [AI] technology has been recognized as a revolutionary concept that allows the integration of complex data, thereby facilitating the scalable extraction of spatial, molecular, and cellular features from routine histopathology and multi-omics platforms. This review of the current evidence on AI-based spatial immunometabolic studies of the tumor microenvironment aims to provide a comprehensive overview of the current evidence, including AI-based spatial immunometabolic studies of the tumor mi-croenvironment, with special reference to digital pathology, spatial transcriptomics, and multimodal data fusion. The current challenges, including data heterogeneity, model interpretability, generalizability, and biological validation, will be discussed. The emerging trends in AI-based spatial immunometabolism, including multimodal foundation models, federated learning, and spatially resolved target discovery, will be discussed. AI-based spatial immunometabolism will be a cornerstone in precision oncology, with the potential to improve patient stratification, therapeutic approaches, and clinical translation.

## 1. Introduction

Cancer, as the National Cancer Institute (NCI) and Centers for Disease Control and Prevention (CDC) have established, is a public health hazard of paramount importance in more than one hundred diverse diseases that develop in most tissues and recur in secondary sites. Far from being successfully cured with advances in medicine, it continues to be one of the foremost causes of disease and death worldwide. Over the past decades, various therapeutic modalities such as chemotherapy, radiotherapy, surgery, and immunotherapy have been developed and employed in the therapy of any kind of cancer. Although the treatments are highly effective in the clinic, they mostly affect the normal tissue, which has been proven to generate hazardous side effects. Therefore, research today focuses on the generation of targeted and selective therapy that is capable of selectively killing cancer cells with minimal loss of normal physiological function [1].

The tumor microenvironment (TME) is a tumorigenic microenvironment with an active role in tumorigenesis and response to therapy. Tumor cells re-engineer their environment by exerting molecular, cellular, and physical changes on host tissue. Immune cells, stroma cells, blood vessels, and the extracellular matrix (ECM) form the microenvironment, which interacts extensively with cancer cells. These interactions support cancer cell survival, invasion, and metastasis throughout the early stages of tumor development. In addition to the low oxygen and acidic tumor microenvironment, the microenvironment also supports angiogenesis, the development of new blood vessels to supply oxygen and nutrients and for the removal of waste, mechanisms for long-term tumor growth and expansion [2].

In addition to the tumor cells, the TME consists of various types of cells such as cancer-associated fibroblasts (CAFs), endothelial cells (ECs), and pericytes, along with other tissue-specific cells whose overall impact affects tumor behavior. Once considered inert and structural components, the latter are now known to actively participate in cancer growth regulation; they have the capacity to suppress or enhance tumor growth. As cancer progresses, the TME becomes an active phase that supports the growth of the tumor by enabling cell proliferation, invasion, and immune escape. It also facilitates conditioning of distant locations for metastasis and whether cancer cells go dormant or get re-activated. Due to its pivotal function in such procedures, the TME has been considered the most important target for the development of new therapeutic solutions to bridge upcoming cellular and molecular mechanisms supporting cancer survival and treatment resistance [3].

The three-dimensional arrangement of cells within a tumor is an important factor in determining how cancer will act after a treatment has been administered. Emerging evidence from spatial multi-omics has demonstrated that the physical spatial structure of tumor, immune, and supportive cells organize into ordered metabolic niches within the tumor. These variations can order spaces to become increasingly resistant to therapy, demonstrating the relevance of spatial context for understanding the unraveling of therapy responses [4]. In stomach cancer, for example, researchers discovered that cancer cells at the periphery of the tumor formed matured immune and metabolic microenvironments. These differentiated areas enable the cells to develop and survive even in nutrient-poor and low oxygen level environments [4]. In the case of lung cancer, specific cell groups containing highly active tumoral cells and granulocytes are associated with decreased sensitivity to immunotherapy and suggest that such cell groups participate in therapy resistance [5]. Systematic meta-analyses of large sets of spatial omics studies show that structurally disorganized cancer cellular architecture, along with its immunologically and metabolically defective processes, is the hidden logic of drug resistance in a broad spectrum of tumors [6]. Probing the dynamics of these cells at high resolution, scientists have discovered that specific signaling pathways and cellular spatial networks cooperate to control tumor adaptation to stress and develop drug-resistance mechanisms [7]. Single-cell and spatial omics profiling has revealed that the cancer cells can reprogram the host’s immune cell metabolism, including macrophages and T cells, and thus suppress its anti-tumor activities [8]. Imaging assays also reveal that these “metabolic alliances” of tumor cells with host cells within a local area build drug-protective microenvironments [9].

Artificial intelligence (AI) in cancer studies on the TME has been flourishing, revolutionizing methods in the exploration of cancer in histopathology, spatial transcriptomics, and metabolomics. In histopathology, AI programs can detect tiny features in cancer tissues, such as tumor budding and cancer-stroma cell interactions, with high precision. For instance, in colorectal cancer, diagnostic systems based on AI have detected more than 97% correctly, which has enabled pathologists to deliver quicker, more precise, and more standardized reports [10]. Advances in deep learning have enabled scientists to transform high-resolution images of tumors in manners previously unachievable with any other tool. These technologies can now identify complex patterns of the immune system and subtle microenvironmental subtleties that are normally missed by visual inspection using conventional methods [11].

Apart from imaging, AI and machine learning in spatial transcriptomics are helping to decide how gene expression is mapped across various parts of a tumor. This approach is providing new understanding of the behavior of the cancer cells and immune cells and how they interact and transform with the addition of therapy [12]. In spatial metabolomics, AI plays the same role in that it deciphers the chemical and energy networks underlying tumor development and the processes by which disrupted metabolism in the TME promotes immunosuppression and drug resistance [13]. Combined, these technologies represent how AI is conjoining the visual, spatial, and molecular aspects of cancer biology to deliver an integrated view of tumor dynamics and response to therapy.

The aim of this review is to evaluate emerging evidence for the application of AI in spatial immunometabolic analysis of the TME. In the current year, cancer research would not be complete without artificial intelligence as an essential ingredient in integrating histopathology, spatial transcriptomics, and metabolomics data. This multi-faceted perspective has enhanced understanding of the intricate cellular and molecular interactions characteristic of cancer and how it reorganizes treatment responses. Though many analyses of AI use in a single field such as digital pathology or spatial genomics have been done, few have attempted to combine these areas of work in an integrated way. This absence denies us information on how metabolic activity and spatial organization collectively control immune behavior and treatment resistance in the TME.

With reciprocal interaction of knowledge from these intersecting fields, the review integrates established methodological advances, defines typical challenges, and suggests innovative research fronts. Enhanced clinician comprehension of AI-based TME profiling can facilitate improved patient stratification, improved evidence-based treatment choice, and improved prediction of therapy response for clinicians. These can be utilized by policymakers for safe and accountable integration of AI technology into precision oncology. This synthesis underpins computational model optimizers, multimodal biomarker validation, and reproducible large-scale spatial data integration. As a response to the rapid expansion of spatial omics and AI-related research post-2023, it is now timely and necessary to review the current literature in order to provide seamless innovations and condense them into firm clinical progress.

A critical aspect of AI-driven spatial analysis lies in its ability to bridge computational outputs with biological mechanisms. Rather than functioning as purely predictive tools, modern AI models, particularly graph neural networks and attention-based architectures, can identify spatially constrained cellular interactions, infer pathway activation states, and reconstruct metabolic dependencies within the tumor microenvironment. For instance, spatial co-localization patterns between glycolytic tumor cells and immunosuppressive macrophages can be computationally learned and linked to lactate-mediated T-cell suppression. Such approaches move beyond correlation and enable hypothesis generation regarding causal immunometabolic mechanisms, positioning AI as a tool for mechanistic discovery rather than solely pattern recognition.

## 2. Tumor Microenvironment: Key Concepts

The TME is the living space around a tumor. It contains blood and lymphatic vessels, fibroblasts, endothelial cells, many types of immune cells, cytokines, extracellular vesicles, and the extracellular matrix. Chemistry and physics matter here as well. Low extracellular pH, pockets of hypoxia, high interstitial fluid pressure, and fibrosis all shape how tumors grow, spread, hide from immunity, and resist therapy. More than a century ago, Stephen Paget described the seed and soil idea. A seed cannot grow on barren ground, and a malignant cell will not thrive unless its neighborhood makes room for it. That is why reading the surroundings of cancer cells and mapping their metabolism can guide treatments that spare healthy tissue. Neighboring cells often shift their metabolic programs in ways that are very different from what they showed before the cancer appeared [14].

Many cell types take part in this. Fibroblasts, endothelial cells, adipocytes, immune cells, and neuroendocrine cells all leave a mark. Acellular players such as the extracellular matrix, extracellular vesicles, and cytokines add their own signals. The physical setting matters too. Low pH, hypoxia, raised interstitial pressure, and fibrosis each push tumors toward progression. Interactions between cells and stroma amplify these signals over time. Cancer-associated fibroblasts sustain proliferative signaling, switch on angiogenesis and metastasis, promote tumor related inflammation, help tumors escape immune attack, reprogram cellular metabolism, and feed genome instability. They are usually tumor promoting, although isolated tumor restraining roles have been noted. They are abundant in the tumor microenvironment and are often identified by alpha smooth muscle actin, FAP alpha, FSP 1 or S100A4, and PDGFR beta. Their exact origins remain debated, and activation can occur through both reactive oxygen and TGF beta pathways [14]. Given the central role of metabolic reprogramming in shaping immune responses within the tumor microenvironment, a focused understanding of immunometabolism is essential. From a spatial perspective, these cell populations exhibit distinct localization patterns within the tumor microenvironment, which directly influence their metabolic interactions and functional roles. Artificial intelligence-driven spatial analysis enables the identification and quantification of these patterns, facilitating the mapping of cell–cell interactions and immunometabolic niches [15].

### Immunometabolism in the Tumor Microenvironment

Immunometabolism describes the bidirectional relationship between cellular metabolic pathways and immune cell function, which collectively determine tumor progression, immune evasion, and therapeutic response.

Cancer cells undergo profound metabolic reprogramming, most notably the Warburg effect, in which glucose is preferentially converted to lactate even in the presence of oxygen. This metabolic shift is not limited to tumor cells but extends to stromal components such as cancer-associated fibroblasts (CAFs), establishing a metabolically coupled ecosystem. Lactate produced in hypoxic tumor regions is exported via monocarboxylate transporter 4 (MCT4) and taken up by oxygenated tumor cells through MCT1, fueling oxidative metabolism. To prevent intracellular acidification, tumor cells employ buffering mechanisms including Na^+^/H^+^ exchanger 1 (NHE1) and carbonic anhydrase IX (CA9), enabling survival under metabolically hostile conditions [16,17].

The accumulation of lactate within the TME has profound immunosuppressive effects. Elevated lactate levels inhibit cytotoxic CD8^+^ T-cell and natural killer (NK) cell activity while promoting regulatory T-cell (Treg) expansion and M2-like macrophage polarization. Hypoxic conditions further exacerbate this environment through stabilization of hypoxia-inducible factor 1-alpha (HIF-1α), which enhances glycolysis, angiogenesis, and expression of immunosuppressive mediators [18,19].

In addition to glucose metabolism, amino acid pathways play a crucial role in immune regulation. Tumor-associated cells frequently deplete arginine and tryptophan through enzymes such as arginase-1 and indoleamine 2,3-dioxygenase (IDO), impairing T-cell proliferation and promoting immune tolerance. Lipid metabolism also contributes to immunometabolic remodeling, where fatty acid oxidation supports regulatory and memory T-cell phenotypes, while lipid accumulation in antigen-presenting cells can impair immune activation [20,21].

Oxidative stress further integrates metabolic and immune signaling. Reactive oxygen species (ROS) generated within tumor and stromal compartments stabilize HIF-1α and modulate signaling pathways that promote tumor survival and immune suppression [20,21,22].

These interconnected pathways highlight that metabolic gradients within the TME are not merely byproducts of tumor growth but active regulators of immune function. Importantly, these processes are spatially organized, forming distinct immunometabolic niches characterized by localized nutrient availability, oxygen levels, and signaling interactions [23,24,25,26].

Understanding these spatially resolved metabolic interactions requires advanced computational approaches. AI has emerged as a powerful tool to decode these complex immunometabolic landscapes. By integrating spatial transcriptomics, metabolomics, and histopathological imaging, AI models can infer metabolic states, identify spatially resolved immune-metabolic interactions, and classify tumor regions into functional niches such as glycolytic, hypoxic, or immune-infiltrated zones [23,24].

Cancer-associated fibroblasts sustain tumor growth through extracellular matrix remodeling, secretion of cytokines, and modulation of immune responses. They promote immune evasion, angiogenesis, and metastatic progression [25].

**Figure 1:**
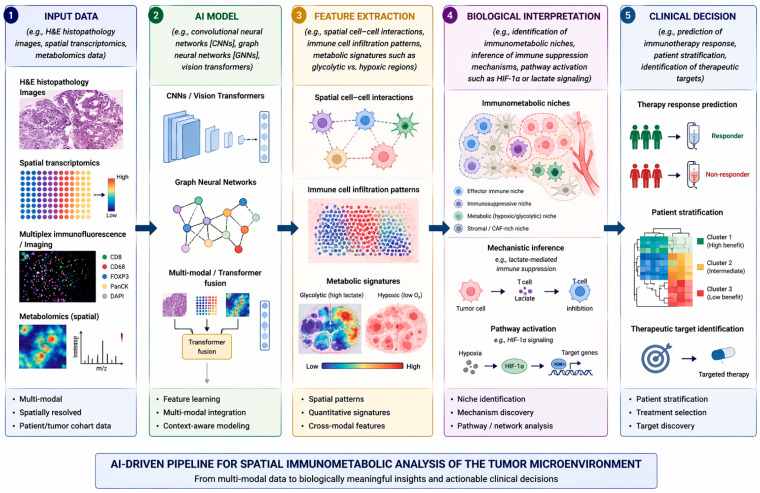
Spatial immunometabolic interactions in the TME.

While these biological components define the tumor microenvironment, understanding their spatial organization and metabolic interactions requires advanced computational approaches. AI-driven spatial analysis provides the framework to quantify these relationships at scale, forming the central focus of this review. While the cellular composition of the TME defines its biological complexity, understanding the spatial organization and metabolic interactions of these components requires advanced computational approaches. AI-driven spatial analysis provides a framework to integrate these features, forming the basis for subsequent analytical and clinical applications [23,24,25,26,27]. These interconnected metabolic and immune interactions are spatially organized within the tumor microenvironment, forming localized immunosuppressive niches that support tumor progression and therapeutic resistance (Figure 1).

## 3. AI in TME Analysis

### 3.1. AI Models Applied to H&E or Multiplexed Images

**Figure 2:**
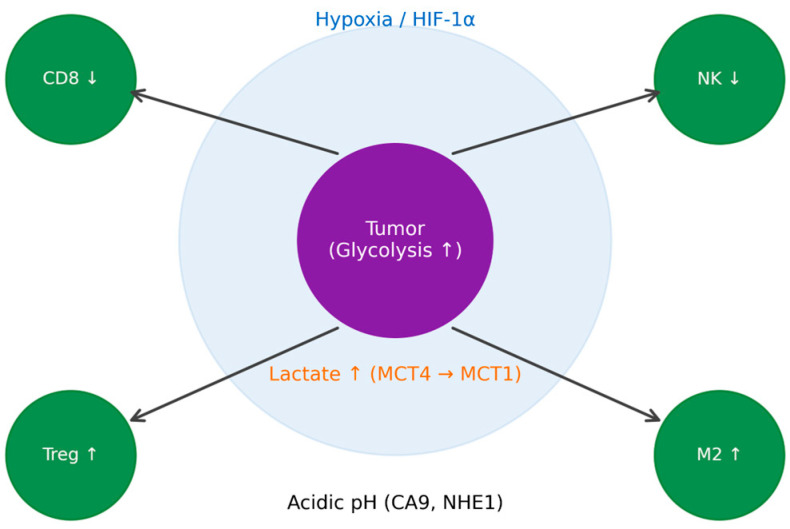
AI pipeline for spatial TME analysis.

AI in histopathology has been shown to be a strong morphologic and molecular discriminator of the TME (Figure 2). Through modeling from run-of-the-mill hematoxylin and eosin [H&E] sections or high-level multiplexed imaging data, these models are able to detect cell population heterogeneity, de-mix spatial relationships, and infer immunometabolic status to therapeutic response. The more recent attempts have been focused on projecting histologic patterns onto molecular and immune maps. Patkar et al. [2024] presented HistoTME, a weakly supervised multi-task attention-based model that, through direct prediction, identifies 30 immune and stromal gene-signature scores from H&E scans of non-small cell lung cancer (NSCLC) [28]. The model achieved a mean Pearson correlation of 0.50 with RNA-seq data and an AUROC of 0.75 for first-line immune checkpoint inhibitor [ICI] response prediction, demonstrating that digital pathology can detect molecular details imperceptible to human vision.

Lapuente-Santana et al. (2024) continued this trend with SPoTLIghT, a transfer-learning approach that integrated H&E images and transcriptomics toward spatial profiling of melanoma [29]. They developed tile-level maps of the probability of cell-type tumor, immune, stromal, and endothelial cells and constructed spatial graphs that quantitatively assessed their interaction. Spatial graph properties were also used to forecast one-year survival with an area under the curve of 0.88, which was an indicator of the ability of AI in reviving the immune architecture of the tumor and identifying patterns of clinical relevance.

In ccRCC, Nyman et al. (2023) used a spatially aware deep learning model that represented histology as a graph structure with neighborhood relationships [30]. The model detected PBRM1 mutation micro-heterogeneity signatures and ICI response heterogeneity and asserted that spatial AI can distinguish histologic correlates of molecular and immunologic heterogeneity.

Other than H&E, imaging mass cytometry multiplexed imaging platforms, spatial transcriptomics, and multiplex immunofluorescence (mIF) provide cell-resolved ground truth information to validate and train AI. Semba et al. (2024) mapped the technologies and related analysis pipelines and outlined the way multiplexed proteomic maps provide molecular ground truthing for computational histology [31]. These in turn enable AI systems to decipher morphological signals into metabolic and immune signatures and enhance explainability. Although digital pathology primarily captures morphological features, recent advances have demonstrated its potential to infer underlying metabolic states. AI models can identify histomorphological patterns associated with glycolysis, hypoxia, and immune infiltration, thereby linking tissue architecture with metabolic activity. Integration with spatial transcriptomics and metabolomics further enhances this capability, enabling correlation between imaging features and functional metabolic pathways.

#### 3.1.1. Accomplishments

Histopathology-informed AI has performed magnificently in the last three years. SPoTLIghT and HistoTME models can make predictions of immune and stromal cell composition to molecular-level accuracy and predict spatial organization with prognostic value [28,29]. Graph-based and attention mechanisms allow models to highlight histologically significant regions and make the model interpretable. These models have been capable of identifying biologically significant features such as tertiary lymphoid structures and spatial immune exclusion that are predictive of response to therapy [29,30].

Scalability might be one of the most important strengths of these approaches. Digital pathology is already a gold standard versus expensive molecular assays or multi-omics. AI can then leverage current H&E collections and reinterpret them to generate population-scale spatially resolved immunometabolic data with unprecedented access for clinical research and translation.

#### 3.1.2. Limitations

Trained to humble accuracies, most of the shortcomings devalue clinical usefulness. One, most of the models are weakly supervised, most frequently outperforming in bulk transcriptomic or slide-level annotation rather than actual single-cell precision [28,29]. Two, staining, scanning, and pre-analytical heterogeneity can ravage reproducibility between centers. Three, most of the research is retrospective cohorts; few have tested prospectively and sought multi-center validation.

Interpretability is also a large concern. While attention maps and heatmaps increase transparency, biological relevance of spatial features generated by AI will tend to have to be correlated against multiplexed imaging or transcriptomics in space [31]. Furthermore, current models focus on immune infiltration at the expense of metabolic states that regulate immune responses, an area that is core to immunometabolism and to drug response.

#### 3.1.3. Predictive Capabilities

One of the most precious uses of AI in histopathology lies in its potential to predict a tumor’s response to immunotherapy. Hu et al. (2021) initially showed that convolutional neural networks were capable of separating responders from non-responders to anti-PD-1 therapy in melanoma and lung cancer (AUC 0.78 and 0.65, respectively) [32]. While small cohorts limited it, this proof-of-concept paper did show that H&E stain images did contain immunologically informative data.

Grounding models, for example HistoTME and the spatially informed Nyman et al. model, achieved improved predictive performance by capturing AI-derived features that condense contextual features e.g., tumor and immune cell spatial co-locality that reflect actual microenvironmental function [28,30]. Particular note should be made of the observation that such AI-derived features are already showing potential to complement traditional biomarkers such as PD-L1 or tumor mutational burden.

The second wave will advance our understanding via the fusion of metabolic markers and multi-plexed imaging. Future systems will predict immunometabolic phenotypes (e.g., glycolytic vs. oxidative niches) and immune constitution from H&E morphology and map spatial immunometabolic patterns to ICI effects. Jiang et al. (2023) showed this promise with a biology-constrained AI model using pathologic and molecular priors to predict prognosis and benefit from immunotherapy in multiple cancer types [33]. The comparison in Table 1 highlights how AI serves as a bridge between widely available histological data and high-resolution spatial omics, enabling scalable biological inference.

### 3.2. Spatial Transcriptomics and AI

#### 3.2.1. Methods for Spatially Resolved Gene Expression

Spatial transcriptomics (ST) has changed how researchers study gene expression in tissues while keeping the spatial context. Broadly, ST technologies fit into two categories: sequencing-based and imaging-based methods. Sequencing-based platforms, such as 10× Genomics Visium, Slide-seq, and High-Definition Spatial Transcriptomics (HDST), capture mRNA from tissue sections using barcoded arrays or beads. These techniques cover the whole genome and are highly scalable. However, each “spot” often contains transcripts from multiple cells, which limits single-cell resolution [34].

In contrast, imaging-based approaches, including MERFISH [Multiplexed Error-Robust Fluorescence In Situ Hybridization], seqFISH+, and CosMx Spatial Molecular Imager, detect fluorescently labeled transcripts directly within intact tissues. These methods offer subcellular precision but usually target a smaller set of genes [35]. To connect the two methods, researchers increasingly combine spatial transcriptomic data with single-cell RNA sequencing (scRNA-seq). This allows them to identify which cell types occupy specific spatial niches, achieving both spatial accuracy and transcriptomic detail.

#### 3.2.2. AI Approaches for Clustering and Spatial Domain Detection

AI and machine learning have greatly improved the analysis of spatial transcriptomic data, especially for spatial clustering and domain detection. Traditional clustering methods often miss spatial continuity, leading to fragmented tissue domains. Recent graph neural network (GNN)-based frameworks, like those suggested by Li et al., integrate spatial coordinates and transcriptomic similarities to pinpoint biologically coherent spatial domains [36].

Self-supervised models such as SpaGIC use contrastive learning to balance the effects of gene expression and spatial closeness. These AI approaches have improved sensitivity to subtle transcriptional gradients. They enable researchers to discover fine tissue boundaries and transitional cellular states that were hard to detect before [37].

#### 3.2.3. Niche Identification

Cells exist in highly specialized microenvironments, or niches, where neighboring cells, the composition of the extracellular matrix, and local signaling gradients shape their actions. Spatial transcriptomics helps identify these cell niches by maintaining both molecular and positional data.

Computational frameworks like NICHES and Niche-DE blend ligand-receptor expression data with spatial positioning to define cellular communication patterns in tissue contexts. NICHES illustrates how cell–cell signaling establishes each niche. Niche-DE identifies genes whose expression varies based on the surrounding cellular environment. These methods have given valuable insights into how gene expression changes with local tissue composition and disease [38,39].

#### 3.2.4. Cell–Cell Interaction Inference

Mapping cell–cell communication (CCC) is a key part of spatial transcriptomic analysis. Methods such as collective optimal transport (COT) and proximity-based interaction modeling deduce biologically significant ligand-receptor signaling events by combining gene expression data with spatial proximity. Cang et al. showed that COT allows for the robust identification of ligand-expressing “sender” cells and receptor-expressing “receiver” cells throughout tissue landscapes. Other frameworks, such as stLearn and SpaTalk, extend this by incorporating histological features and graph-based learning [40]. Together, these computational innovations show how signaling networks influence tissue organization in processes like immune infiltration, tumor invasion, and organ development.

### 3.3. Metabolomics and Multi-Omics Integration

Unlike transcriptomics and proteomics, metabolomics provides a direct functional readout of cellular activity by capturing small-molecule metabolites that reflect real-time biochemical processes. This makes it uniquely suited for studying immunometabolism. However, the integration of spatial metabolomics data remains challenging due to technical variability, limited spatial resolution, and the complexity of metabolite identification. AI approaches have begun to address these challenges by enabling data integration, noise reduction, and pattern recognition across spatially resolved datasets. These methods facilitate the identification of metabolic gradients and localized immunometabolic interactions, providing a more comprehensive understanding of tumor biology. Liquid chromatography with mass spectrometry has been the lead platform used to find and validate protein biomarkers in bulk. It analyzes and detects thousands of proteins and variants on a direct level from plasma, urine, or tissue. Two major modes are employed. Global proteomics compares samples via label-free quantitation or isotope labeling such as SILAC, 18O, dimethyl tagging, iTRAQ, or TMT to identify candidate biomarkers throughout the proteome but may cut very low-abundance signals. Targeted proteomics is quantitative measurement of predefined proteins by selected or parallel reaction monitoring and data-independent acquisition using stable isotope standards for accurate quantification. Because protein levels in biofluids cover very broad ranges, enrichment methods such as immunodepletion, immunoprecipitation, antibody-peptide capture coupled to antibodies, strong cation exchange, or hydrophilic interaction chromatographic separations are used most frequently to enhance sensitivity [41]. New recent developments in high-pressure reversed-phase separation with smart fraction selection now make it possible to detect biomarkers even at very low abundance in clinical samples. The pervasive need remains high-throughput sensitivity for low-abundance protein detection in a robust manner. Uniform treatment and sample preparation are crucial, with the potential to impact data quality through factors such as fixation and storage. Despite this, proteomic profiling, mass spectrometry imaging, and artificial intelligence-driven analysis are driving stepwise toward clinical decision support from discovery, enabling earlier diagnosis, enhanced subtyping, enhanced therapy matching, and identification of new therapeutic targets [42].

The recent rapid growth in checkpoint therapies further revolutionized cancer therapy but are still limited in the clinic by heterogeneous response and immune-related toxicities. As a process complementary to proteomics, artificial intelligence elucidates how and why patients respond heterogeneously through transcriptomic analysis. Bulk RNA sequencing measures average gene expression, but single-cell RNA sequencing deciphers the heterogeneous tumor and stromal cell population at single-cell resolution. Machine learning and AI pipelines deconvolve bulk profiles, register datasets, and denoise to uncover latent biological patterns such as lineage plasticity and copy number alterations. As a result of these advances, scientists are now able to map intratumor heterogeneity, monitor immune tumor dynamics, and describe cellular features associated with disease progression, patient prognosis, and treatment response [32,42].

Clinically, deep learning algorithms trained on single-cell transcriptomes have identified T-cell subsets that are predictive of response to immunotherapy, e.g., CXCL13+ and XCL1 or XCL2+ cells, and have revealed stroma programs suppressing immunity, e.g., fibroblast and macrophage checkpoint resistance states. Artificial intelligence-driven predictive models constructed with bulk and single-cell data now are able to accurately predict patient response, relapse, or resistance and have also uncovered actionable pathways involving IRAK4, NKG7, ELF3, SHP2, and CSF1R. The same data also uncovered mechanisms of immune-related toxicities and associated with various CD8 effector programs, tumor necrosis factor signaling, mTOR activation, and tissue-specific myeloid circuits with side effects including colitis, hepatitis, myocarditis, and arthritis. The integration of AI-aided transcriptomic, proteomic, and spatial information now holds the promise for ever more precise cancer medicine. Combined, they offer a multimodal architecture vision of tumor dynamics that enables enhanced prediction of response to treatment, guiding combination regimens to prevent resistance, and enabling safer, more personalized immunotherapy [43].

### 3.4. Evaluation of AI Models in Spatial Immunometabolism

Robust evaluation of AI models is critical for enabling their reliable clinical translation. Contemporary approaches employ a range of architectures, including CNNs, vision transformers (ViTs), and graph neural networks (GNNs), each tailored to specific data modalities such as histopathological images or spatially resolved omics data. The selection of model architecture plays a pivotal role in determining the ability to capture spatial dependencies, hierarchical features, and complex biological interactions.

Despite these advances, bias remains a significant challenge. Models developed using single-center datasets are prone to learning institution-specific staining characteristics or population-specific features, thereby limiting their generalizability. Additional sources of bias, including class imbalance, selection bias, and annotation bias, can further compromise model robustness and lead to misleading conclusions.

Benchmarking of AI models in this domain is further constrained by the absence of standardized datasets and evaluation frameworks. In contrast to fields such as computer vision, where large-scale benchmarks like ImageNet have facilitated consistent model comparison, spatial omics lacks universally accepted reference datasets. Consequently, performance metrics such as the area under the receiver operating characteristic curve (AUROC), spatial correlation coefficients, and survival prediction accuracy are applied inconsistently across studies, hindering reproducibility and comparability.

Future efforts should prioritize the establishment of rigorous validation strategies, including multi-center studies, external validation cohorts, and the development of open-access benchmark datasets. Furthermore, integrating biological validation of model predictions will be essential to ensure that computational outputs are both mechanistically meaningful and clinically actionable.

## 4. AI for Predicting Therapy

### 4.1. AI Models Linking TME Features to Immunotherapy Response

The impact of the TME on immunotherapy efficacy can be increasingly explored using AI-driven approaches, although current models remain limited in fully capturing the underlying biological complexity. Rather than functioning as isolated methodologies, AI techniques in spatial immunometabolism are increasingly applied within integrated analytical frameworks. These frameworks combine multiple model architectures, including convolutional neural networks for image analysis, graph neural networks for modeling spatial relationships, and transformer-based models for multimodal data integration. In addition, explainable AI approaches are incorporated to enhance interpretability and biological relevance. Such integration enables the simultaneous analysis of spatial structure, molecular features, and cellular interactions, providing a comprehensive understanding of tumor biology. The intricacy of immune interactions within tumors is only partially explained by conventional indicators of PD-L1 expression and tumor mutational burden. Recent advances in AI algorithms use molecular, radiological, and histopathological data to describe TME characteristics, enabling more accurate immunotherapy response prediction. For instance, Patkar et al. [2024] created HistoTME, a deep learning model that predicts the response of patients with NSCLC to immunotherapy by extracting spatial immune and stromal cell patterns, from routine hematoxylin and eosin (H&E) slides. In independent validation cohorts, the model outperformed conventional biomarkers with an area under the receiver operating characteristic curve [AUROC] of roughly 0.75 and an accurate estimation of the TME composition [28]. In a similar vein, Jiang et al. [2023] developed a biology-guided deep learning technique that predicts prognosis and immunotherapy response for various cancers by combining transcriptomic and morphological data [33]. These models demonstrate how AI can transform routine pathology data into useful immune activity maps.

#### 4.1.1. Examples of Limitations and Successes

Although most AI models are still in the proof-of-concept stage, a number of them have already predicted patient outcomes following ICI.

The primary achievements have been the discovery of obscure molecular and spatial patterns connected to immune response. Hu et al. (2021), for instance, demonstrated that convolutional neural networks trained from H&E images of patients with lung cancer and melanoma could predict anti-PD-1 response with encouraging accuracy [32]. In keeping with this, Ye et al. (2024) confirmed the potential of imaging-based AI biomarkers by establishing a multimodal CT-based deep learning biomarker predictive of pathological complete response in NSCLC patients treated with neoadjuvant immunochemotherapy [44]. However, small, retrospective cohorts frequently limit the broader generalizability of such successes. Most studies continue to be correlational rather than causal, associating particular traits with results without elucidating the underlying mechanisms. Furthermore, a persistent issue with these models is their interpretability. Even when predictive performance is high, the biological basis for AI-driven decisions is too often ambiguous, which hinders clinical adoption, as Jiang et al. (2023) and others have emphasized [33].

#### 4.1.2. Emerging Trends: Multimodal Models, Graph Neural, and Explainable AI

With explainable models, graph-based inference, and multimodal fusion, the newest developments in AI are well-positioned to tackle these issues. Since each modality captures distinct biological aspects of the TME, Li et al. (2024) discovered that combining multi-omics datasets such as transcriptomics, proteomics, and genomics into AI models significantly increases the accuracy of immunotherapy response prediction [45].

The application of GNNs to the spatial and molecular interactions between immune cells and tumors is another area of interest. Jiang et al. (2024) introduced IRNet, which interprets intercellular signaling for immune activation and resistance using pathway-informed GNNs [46]. Similar to this, Gogoshin and Rodin (2023) have looked into the possibility of using GNNs to represent the TME as a dynamic network, which would enable a more thorough investigation of immune-tumor interactions [47].

Lastly, in order to gain clinical trust, explainable AI [XAI] is being pursued. By using techniques such as concept-bottleneck models and SHAP (SHapley Additive Explanations), researchers can determine which biological characteristics are driving model predictions. The COMPASS framework (Shen et al., 2025) is one such strong example, integrating multimodal foundation models with interpretable latent representations to predict immunotherapy response across various cancer types [48].

## 5. Challenges and Limitations

Despite the rapid advancement in AI-driven spatial and immunometabolic analysis of the TME, several challenges continue to limit its potential.

Data limitations continue to be a major challenge. Many existing studies rely on small sample sizes, often drawn from single institutions or focused on specific cancer types, which reduces their statistical strength and limits how broadly their results can be applied [4,10,11]. Dataset heterogeneity caused by differences in imaging platforms, sequencing methods, and data processing workflows creates major batch effects that can skew biological interpretations and make it difficult to compare results across studies [5,6]. Combining multi-omics data such as spatial transcriptomics, proteomics, and metabolomics adds another layer of complexity, as these methods differ in spatial resolution, molecular depth, and tissue preparation techniques [7,8].

From a model development perspective, key challenges continue to revolve around explainability, generalizability, and reproducibility. While deep learning systems have demonstrated impressive capabilities, they often function as “black boxes,” offering limited insight into the biological reasoning behind their outputs [10,11]. Moreover, the absence of standardized validation frameworks and publicly accessible benchmarks makes it difficult to achieve consistent reproducibility across independent studies [12]. Another limitation is that many AI models are trained on data from a single tumor type or imaging modality, which constrains their ability to perform reliably across diverse patient populations and cancer subtypes [13,49].

At the biological level, our understanding of how immune and metabolic processes interact within the TME is still developing. While spatial multi-omics has begun to shed light on how these networks contribute to treatment resistance, much remains to be uncovered [4,7,9]. The full spectrum of these interactions, particularly how localized metabolic gradients influence immune cell behavior, remains incompletely characterized [2,3]. This gap reduces both the biological interpretability of AI-generated findings and the capacity to confirm computational predictions through functional studies. Additional sources of failure in AI-driven spatial and multi-omics analyses must be carefully considered. Batch effects, arising from variations in sample preparation, sequencing platforms, and staining protocols, can introduce significant technical noise and distort model outputs. Furthermore, many models rely on weak supervision, often using slide-level or bulk annotations rather than single-cell resolution labels, thereby limiting biological precision and interpretability. Generalizability remains another critical concern, as models trained on specific tumor types, cohorts, or institutions frequently exhibit reduced performance when applied to external datasets. In addition, the high dimensionality of multi-omics data increases the risk of overfitting, where models capture dataset-specific noise rather than true underlying biological signals. Addressing these challenges requires the implementation of standardized preprocessing pipelines, robust normalization strategies, domain adaptation techniques, and rigorous external validation to ensure reproducibility and clinical applicability.

Addressing the challenges summarized in Table 2 will require access to larger, multi-center datasets developed under standardized protocols, along with transparent model design and active collaboration between computational scientists, pathologists, and immunologists. Only through such joint efforts can AI-driven spatial immunometabolic analysis progress from exploratory research toward clinically meaningful and applicable tools in cancer management.

## 6. Future Directions

Recent technological advancement has provided the option of simultaneous probing of spatial transcriptomes and metabolomes in intact tissue. Vicari et al. have developed a spatial multimodal analysis pipeline combining histology, MALDI mass spectrometry imaging, and spatial transcriptomics to obtain transcript–metabolite co-variation within the same section [50]. This integration provides unprecedented insight into how gene function is coordinated with local metabolite conditions, paving the way for multimodal AI that is capable of learning complex interactions between morphology, gene expression, and metabolite concentrations. These kinds of AI models, particularly graph neural networks and attention models, are able to read a tissue site as a node in a spatially organized biological graph. Beyond classification, they can generate interpretable embeddings describing coordinated biological functions across molecular layers. Latent spaces so derived might be employed to discover yet unrecognized immunometabolic “niches” in which immune infiltration and metabolic flux together define disease course [50].

With the increasing entry of AI into biological interpretation, there is an increasing necessity for transparency. Toussaint et al. comprehensively presented XAI application in omics, listing techniques such as SHAP, LIME, and intrinsically interpretable neural structures [51]. The article emphasized causal and text-based accounts that transform computational patterns into biologically interpretable hypotheses. Upcoming explainable multimodal models might annotate some areas of tissue with mechanistic claims such as: “Immune suppression forecasted due to high CD73-mediated adenosine and low CXCL9 expression.” These local rationales make transform AI from a diagnostic instrument into a mechanistic partner, capable of formulating hypotheses and reasoning biologically.

One of the largest problems with spatial multi-omics is that data are fragmented between centers with their own machines, tissue types, and privacy boundaries. Federated learning [FL] is a remedy for this that trains global models on decentralized data without exchanging raw files. Ankolekar et al. surveyed FL application in oncology, proving that models trained across several institutions can match or surpass centralized performance with data privacy preserved [52]. If used in spatial omics, federated approaches would bring data from different platforms like Visium, CosMx, MIBI, or MALDI-MSI into alignment and build a global model with underlying universal biological patterns. However, harmonization layers and reproducibility principles must be implemented to prevent batch effects and provide reproducibility across sites.

A key promise of multimodal AI is the identification of new therapeutic targets through the discovery of spatially discrete immunometabolic microenvironments. Tang et al. investigated clear cell renal cell carcinoma (ccRCC) and showed that metabolic heterogeneity co-evolves with immune cell composition to generate distinctive immunometabolic niches that are associated with therapeutic response [53]. They demonstrated that RNA expression can predict local metabolite profiles, in association with metabolic states and immune modulation. Founded on such observations, AI models would be capable of demarcating tissue regions with specific metabolic needs, lactate accumulation, or fatty-acid oxidation that inhibit immune responsiveness. This would allow precise targeting of metabolic pathways in targeted microregions, advancing spatially resolved therapeutic design.

With robust standards lacking, multimodal AI capability is scattered. Khan et al. reviewed spatial transcriptomics alignment and integration methods, urging shared benchmarking datasets, open pipelines, and reproducible benchmarking metrics. Their recommendations are the foundation of harmonized cross-platform analysis and federated research collaborations. The creation of open benchmark repositories, akin to ImageNet for visual computing, could unify the field. Universal metrics, such as spot-wise accuracy of predictions or cross-site agreement, would enable fair comparison among models and drive progress across the community [54].

For clinical translation, AI-driven spatial immunometabolic analysis holds significant promise in advancing precision oncology. By integrating spatial and molecular data, these approaches enable patient stratification based on distinct immunometabolic phenotypes and improve the prediction of immunotherapy response beyond conventional biomarkers such as PD-L1. Furthermore, they facilitate the identification of spatially localized therapeutic targets and support the rational design of combination treatment strategies, including the integration of metabolic interventions with immune checkpoint inhibition. Despite these advances, successful incorporation into clinical workflows requires rigorous regulatory validation, enhanced model interpretability, and confirmation through prospective clinical trials to ensure reliability, safety, and clinical utility. Future research should focus on improving the robustness, interpretability, and generalizability of AI models in spatial oncology. Collaborative efforts between computational scientists, biologists, and clinicians will be essential to ensure that models are both technically sound and biologically meaningful. The development of standardized datasets and benchmarking frameworks will further enhance reproducibility and comparability across studies. Additionally, integrating multimodal data, including genomics, transcriptomics, metabolomics, and imaging, will enable a more holistic understanding of tumor biology. Cross-disciplinary collaborations and prospective clinical validation studies will be critical to accelerate the translation of AI-driven insights into routine clinical decision-making.

## 7. Conclusions

The field of AI is revolutionizing the understanding of the TME at an unprecedented pace by integrating spatial, immune, and metabolic data to create coherent models that can be clinically interpreted. Today, evidence supports the fact that AI can extract prognostic and predictive information from routine histology and spatial multi-omics, thereby revealing immunometabolic niches that control therapy response and resistance. However, to use this technology on a broader scale, data standards, model transparency, and model explanations, along with multi-center validation, are necessary. While AI-driven spatial analysis has demonstrated significant potential in advancing our understanding of tumor immunometabolism, several challenges remain. Future efforts should prioritize the development of interpretable models, robust validation strategies, and clinically relevant endpoints. Importantly, the integration of AI into clinical practice will require not only technological advancements but also interdisciplinary collaboration and regulatory alignment. With continued innovation and validation, AI has the potential to transform the landscape of precision oncology by enabling more accurate diagnosis, prognosis, and therapeutic decision-making.

## Figures and Tables

**Table 1:** Histological Data and Spatial Omics analyzed using AI.

Feature	Histology (H&E)	Spatial Omics
Resolution	Cellular morphology	Molecular + spatial
Cost	Low	High
Availability	Widely available	Limited
Data type	Visual	Transcriptomic/metabolic
AI role	Feature extraction	Data integration
Limitation	Indirect biology	Expensive + complex

**Table 2:** Key Challenges and Limitations in AI-Driven Spatial Immunometabolic Analysis of the TME.

Category	Specific Challenges	Description/Examples
Data-Related	Small sample sizes	Many studies use limited patient cohorts or single-institution datasets, reducing statistical power and external validity [4,10,11].
	Data heterogeneity	Variability in tissue processing, imaging platforms, and sequencing protocols introduces batch effects that confound model performance [5,6].
	Multi-omics integration complexity	Differences in spatial resolution and molecular coverage between transcriptomic, proteomic, and metabolomic datasets complicate integration [7,8].
Model-Related	Explainability [black-box issue]	Deep learning models often lack transparency, making biological interpretation of predictions difficult [10,11].
	Generalizability	AI models trained on a single cancer type or data source may not perform consistently across diverse tumors or populations [13,49].
	Reproducibility	Absence of standardized validation frameworks and shared benchmarks limits reproducibility across studies [12].
Biological	Incomplete understanding of immune-metabolic crosstalk	The interactions between metabolic gradients and immune cell behavior within the TME remain poorly characterized [2,3].
	Limited functional validation	Computational predictions are often not followed by in vitro or in vivo validation to confirm biological mechanisms [4,7,9].

***Abbreviations***: AI, artificial intelligence; TME, tumor microenvironment.

## Data Availability

No new data were created or analyzed in this study.

## Abbreviations

The following abbreviations are used in this manuscript:

AI	Artificial Intelligence
TME	Tumor Microenvironment
NCI	National Cancer Institute
CDC	Centers for Disease Control and Prevention
ECM	Extracellular Matrix
CAFs	Cancer-Associated Fibroblasts
ECs	Endothelial Cells
HIF-1	Hypoxia-Inducible Factor 1
GLUT1	Glucose Transporter 1
GLUT3	Glucose Transporter 3
MCT1	Monocarboxylate Transporter 1
MCT4	Monocarboxylate Transporter 4
LDHA	Lactate Dehydrogenase A
PKM2	Pyruvate Kinase M2
NHE1	Na^+^/H^+^ Exchanger 1
CA9	Carbonic Anhydrase 9
TIGAR	TP53-Induced Glycolysis and Apoptosis Regulator
ROS	Reactive Oxygen Species
TILs	Tumor-Infiltrating Lymphocytes
MDSC	Myeloid-Derived Suppressor Cells
Treg	Regulatory T Cells
CCC	Cell–Cell Communication
GM-CSF	Granulocyte-Macrophage Colony-Stimulating Factor
G-CSF	Granulocyte Colony-Stimulating Factor
M-CSF	Macrophage Colony-Stimulating Factor
TGF-β	Transforming Growth Factor Beta
TNF-α	Tumor Necrosis Factor Alpha
VEGF	Vascular Endothelial Growth Factor
FGF	Fibroblast Growth Factor
PDGF	Platelet-Derived Growth Factor
IL	Interleukin
CCL	C-C Motif Chemokine Ligand
CXCL	C-X-C Motif Chemokine Ligand
CCR	Chemokine Receptor
CXCR	C-X-C Chemokine Receptor
COX2	Cyclooxygenase-2
PD-1	Programmed Cell Death Protein 1
PD-L1	Programmed Death-Ligand 1
CTLA-4	Cytotoxic T-Lymphocyte–Associated Protein 4
ICI	Immune Checkpoint Inhibitor
NSCLC	Non-Small Cell Lung Cancer
ccRCC	Clear Cell Renal Cell Carcinoma
CRC	Colorectal Cancer
HCC	Hepatocellular Carcinoma
H&E	Hematoxylin and Eosin
mIF	Multiplex Immunofluorescence
ST	Spatial Transcriptomics
scRNA-seq	Single-Cell RNA Sequencing
RNA-seq	RNA Sequencing
MERFISH	Multiplexed Error-Robust Fluorescence In Situ Hybridization
HDST	High-Definition Spatial Transcriptomics
MALDI-MSI	Matrix-Assisted Laser Desorption/Ionization Mass Spectrometry Imaging
LC-MS	Liquid Chromatography–Mass Spectrometry
GNN	Graph Neural Network
XAI	Explainable Artificial Intelligence
SHAP	SHapley Additive Explanations
AUROC	Area Under the Receiver Operating Characteristic Curve
AUC	Area Under the Curve
FL	Federated Learning
SILAC	Stable Isotope Labeling by Amino Acids in Cell Culture
iTRAQ	Isobaric Tags for Relative and Absolute Quantification
TMT	Tandem Mass Tags
PFKFB3	6-Phosphofructo-2-Kinase/Fructose-2,6-Bisphosphatase 3
EVs	Extracellular Vesicles
MMP	Matrix Metalloproteinase

## References

[1] Erasha A.M., EL-Gendy H., Aly A.S., Fernández-Ortiz M., Sayed R.K.A. (2025). The Role of the Tumor Microenvironment [TME] in Advancing Cancer Therapies: Immune System Interactions, Tumor-Infiltrating Lymphocytes [TILs], and the Role of Exosomes and Inflammasomes. Int. J. Mol. Sci..

[2] Anderson N.M., Simon M.C. (2020). The tumor microenvironment. Curr. Biol..

[3] De Visser K.E., Joyce J.A. (2023). The evolving tumor microenvironment: From cancer initiation to metastatic outgrowth. Cancer Cell.

[4] Sun C., Wang A., Zhou Y., Chen P., Wang X., Huang J., Gao J., Wang X., Shu L., Lu J. (2023). Spatially resolved multi-omics highlights cell-specific metabolic remodeling and interactions in gastric cancer. Nat. Commun..

[5] Aung T.N., Monkman J., Warrell J., Vathiotis I., Bates K.M., Gavrielatou N., Trontzas I.P., Tan C.W., Fernandez A.I., Moutafi M. (2025). Spatial signatures for predicting immunotherapy outcomes using multi-omics in non-small cell lung cancer. Nat. Genet..

[6] Zhang Y., Yang C., Chen X., Wu L., Yuan Z., Zhang F., Qian B. (2025). Cancer therapy resistance from a spatial-omics perspective. Clin. Transl. Med..

[7] Du Y., Ding X., Ye Y. (2024). The spatial multi-omics revolution in cancer therapy: Precision redefined. Cell Rep. Med..

[8] Zhang C.C., Feng H.R., Zhu J., Hong W.F. (2025). Application of spatial and single-cell omics in tumor immunotherapy biomarkers. LabMed Discov..

[9] Chen P., Geng H., Ma B., Zhang Y., Zhu Z., Li M., Chen S., Wang X., Sun C. (2025). Integrating spatial omics and single-cell mass spectrometry imaging reveals tumor–host metabolic interplay in hepatocellular carcinoma. Proc. Natl. Acad. Sci. USA.

[10] Lobanova O.A., Kolesnikova A.O., Ponomareva V.A., Vekhova K.A., Shaginyan A.L., Semenova A.B., Nekhoroshkov D.P., Kochetkova S.E., Kretova N.V., Zanozin A.S. (2024). Artificial intelligence [AI] for tumor microenvironment [TME] and tumor budding [TB] identification in colorectal cancer [CRC] patients: A systematic review. J. Pathol. Inform..

[11] Xie T., Huang A., Yan H., Ju X., Xiang L., Yuan J. (2024). Artificial intelligence: Illuminating the depths of the tumor microenvironment. J. Transl. Med..

[12] Feng S., Yin X., Shen Y. (2025). Artificial intelligence-powered precision: Unveiling the tumor microenvironment for a new frontier in personalized cancer therapy. Intell. Med..

[13] Chon H.J., Kim G., Kang B., Hong J.Y., Kang H., Hwang S., Lee S.H., Jung S., An C., Lee W.S. (2024). Artificial intelligence [AI]-powered tumor microenvironment [TME] analysis to identify potential biomarkers for ICIs with or without bevacizumab in hepatocellular carcinoma [HCC]. J. Clin. Oncol..

[14] Wei R., Liu S., Zhang S., Min L., Zhu S. (2020). Cellular and Extracellular Components in Tumor Microenvironment and Their Application in Early Diagnosis of Cancers. Anal. Cell. Pathol..

[15] Sazeides C., Le A., Le A. (2021). Metabolic Relationship Between Cancer-Associated Fibroblasts and Cancer Cells. The Heterogeneity of Cancer Metabolism.

[16] Uribe-Querol E., Rosales C. (2015). Neutrophils in Cancer: Two Sides of the Same Coin. J. Immunol. Res..

[17] Malfitano A.M., Pisanti S., Napolitano F., Di Somma S., Martinelli R., Portella G. (2020). Tumor-Associated Macrophage Status in Cancer Treatment. Cancers.

[18] Farhood B., Najafi M., Mortezaee K. (2019). CD8+ cytotoxic T lymphocytes in cancer immunotherapy: A review. J. Cell. Physiol..

[19] Ohue Y., Nishikawa H. (2019). Regulatory T [Treg] cells in cancer: Can Treg cells be a new therapeutic target?. Cancer Sci..

[20] Umansky V., Blattner C., Gebhardt C., Utikal J. (2016). The Role of Myeloid-Derived Suppressor Cells [MDSC] in Cancer Progression. Vaccines.

[21] Lee H., Hong I. (2017). Double-edged sword of mesenchymal stem cells: Cancer-promoting versus therapeutic potential. Cancer Sci..

[22] Hida K., Maishi N., Takeda R., Hida Y., Segi C.M. (2022). The Roles of Tumor Endothelial Cells in Cancer Metastasis. Metastasis.

[23] Oronsky B., Ma P.C., Morgensztern D., Carter C.A. (2017). Nothing But NET: A Review of Neuroendocrine Tumors and Carcinomas. Neoplasia.

[24] Fouladzadeh A., Dorraki M., Min K.K.M., Cockshell M.P., Thompson E.J., Verjans J.W., Allison A., Bonder C.S., Abbott D. (2021). The development of tumour vascular networks. Commun. Biol..

[25] Kalluri R., McAndrews K.M. (2023). The role of extracellular vesicles in cancer. Cell.

[26] Popova N.V., Jücker M. (2022). The Functional Role of Extracellular Matrix Proteins in Cancer. Cancers.

[27] Yuan Y. (2016). Spatial Heterogeneity in the Tumor Microenvironment. Cold Spring Harb. Perspect. Med..

[28] Patkar S., Chen A., Basnet A., Bixby A., Rajendran R., Chernet R., Faso S., Kumar P.A., Desai D., El-Zammar O. (2024). Predicting the tumor microenvironment composition and immunotherapy response in non-small cell lung cancer from digital histopathology images. npj Precis. Oncol..

[29] Lapuente-Santana Ó., Kant J., Eduati F. (2024). Integrating histopathology and transcriptomics for spatial tumor microenvironment profiling in a melanoma case study. npj Precis. Oncol..

[30] Nyman J., Denize T., Bakouny Z., Labaki C., Titchen B.M., Bi K., Hari S.N., Rosenthal J., Mehta N., Jiang B. (2023). Spatially aware deep learning reveals tumor heterogeneity patterns that encode distinct kidney cancer states. Cell Rep. Med..

[31] Semba T., Ishimoto T. (2024). Spatial analysis by current multiplexed imaging technologies for the molecular characterisation of cancer tissues. Br. J. Cancer.

[32] Hu J., Cui C., Yang W., Huang L., Yu R., Liu S., Kong Y. (2021). Using deep learning to predict anti-PD-1 response in melanoma and lung cancer patients from histopathology images. Transl. Oncol..

[33] Jiang Y., Zhang Z., Wang W., Huang W., Chen C., Xi S., Ahmad M.U., Ren Y., Sang S., Xie J. (2023). Biology-guided deep learning predicts prognosis and cancer immunotherapy response. Nat. Commun..

[34] Chen T.Y., You L., Hardillo J.A.U., Chien M.P. (2023). Spatial Transcriptomic Technologies. Cells.

[35] Eng C.-H.L., Lawson M., Zhu Q., Dries R., Koulena N., Takei Y., Yun J., Cronin C., Karp C., Yuan G.-C. (2019). Transcriptome-scale super-resolved imaging in tissues by RNA seqFISH+. Nature.

[36] Li J., Chen S., Pan X., Yuan Y., Shen H.B. (2022). Cell clustering for spatial transcriptomics data with graph neural networks. Nat. Comput. Sci..

[37] Liu W., Wang B., Bai Y., Liang X., Xue L., Luo J. (2024). SpaGIC: Graph-informed clustering in spatial transcriptomics via self-supervised contrastive learning. Brief. Bioinform..

[38] Raredon M.S.B., Yang J., Kothapalli N., Lewis W., Kaminski N., Niklason L.E., Kluger Y. (2023). Comprehensive visualization of cell–cell interactions in single-cell and spatial transcriptomics with NICHES. Bioinformatics.

[39] Mason K., Sathe A., Hess P.R., Rong J., Wu C.-Y., Furth E., Susztak K., Levinsohn J., Ji H.P., Zhang N. (2024). Niche-DE: Niche-differential gene expression analysis in spatial transcriptomics data identifies context-dependent cell-cell interactions. Genome Biol..

[40] Cang Z., Zhao Y., Almet A.A., Stabell A., Ramos R., Plikus M.V., Atwood S.X., Nie Q. (2023). Screening cell–cell communication in spatial transcriptomics via collective optimal transport. Nat. Methods.

[41] Berghmans E., Boonen K., Maes E., Mertens I., Pauwels P., Baggerman G. (2020). Implementation of MALDI Mass Spectrometry Imaging in Cancer Proteomics Research: Applications and Challenges. J. Pers. Med..

[42] Wang H., Shi T., Qian W.-J., Liu T., Kagan J., Srivastava S., Smith R.D., Rodland K.D., Camp D.G. (2016). The clinical impact of recent advances in LC-MS for cancer biomarker discovery and verification. Expert Rev. Proteom..

[43] Gui Y., He X., Yu J., Jing J. (2023). Artificial Intelligence-Assisted Transcriptomic Analysis to Advance Cancer Immunotherapy. J. Clin. Med..

[44] Ye G., Wu G., Qi Y., Li K., Wang M., Zhang C., Li F., Wee L., Dekker A., Han C. (2024). Non-invasive multimodal CT deep learning biomarker to predict pathological complete response of non-small cell lung cancer following neoadjuvant immunochemotherapy: A multicenter study. J. Immunother. Cancer.

[45] Li Y., Wu X., Fang D., Luo Y. (2024). Informing immunotherapy with multi-omics driven machine learning. npj Digit. Med..

[46] Jiang Y., Immadi M.S., Wang D., Zeng S., Chan Y.O., Zhou J., Xu D., Joshi T. (2025). IRnet: Immunotherapy response prediction using pathway knowledge-informed graph neural network. J. Adv. Res..

[47] Gogoshin G., Rodin A.S. (2023). Graph Neural Networks in Cancer and Oncology Research: Emerging and Future Trends. Cancers.

[48] Shen W., Nguyen T.H., Li M.M., Huang Y., Moon I., Nair N., Marbach D., Zitnik M. (2025). Generalizable AI predicts immunotherapy outcomes across cancers and treatments. Pharmacol. Ther..

[49] Kim H., Choi J.H., Lim Y., Yoon S.J., Jang K.-T., Ock C.-Y., Choi Y.H., Joe C., Song S., Moon J. (2025). Artificial Intelligence–Powered Spatial Analysis of Immune Phenotypes in Resected Pancreatic Cancer. JAMA Surg..

[50] Vicari M., Mirzazadeh R., Nilsson A., Shariatgorji R., Bjärterot P., Larsson L., Lee H., Nilsson M., Foyer J., Ekvall M. (2024). Spatial multimodal analysis of transcriptomes and metabolomes in tissues. Nat. Biotechnol..

[51] Toussaint P.A., Leiser F., Thiebes S., Schlesner M., Brors B., Sunyaev A. (2023). Explainable artificial intelligence for omics data: A systematic mapping study. Brief. Bioinform..

[52] Ankolekar A., Boie S., Abdollahyan M., Gadaleta E., Hasheminasab S.A., Yang G., Beauville C., Dikaios N., Kastis G.A., Bussmann M. (2025). Advancing breast, lung and prostate cancer research with federated learning. A systematic review. npj Digit. Med..

[53] Tang C., Xie A.X., Liu E.M., Kuo F., Kim M., DiNatale R.G., Golkaram M., Chen Y.-B., Gupta S., Motzer R.J. (2023). Immunometabolic coevolution defines unique microenvironmental niches in ccRCC. Cell Metab..

[54] Khan M., Arslanturk S., Draghici S. (2025). A comprehensive review of spatial transcriptomics data alignment and integration. Nucleic Acids Res..

